# Artificial intelligence-assisted chest X-ray for tuberculosis case finding in low- and middle-income countries: implementation experiences and impact

**DOI:** 10.1093/bjro/tzag007

**Published:** 2026-03-13

**Authors:** Shibu Vijayan, Aswathy M Nair, Nimmy Dominic, Justy Antony Chiramal, Saniya Pawar, Latashree Goswami

**Affiliations:** Global Health Innovation, Clinical, and Operational Research Division, Qure.ai Technologies Pvt Ltd, Bengaluru, Karnataka, 560042, India; Global Health Innovation, Clinical, and Operational Research Division, Qure.ai Technologies Pvt Ltd, Bengaluru, Karnataka, 560042, India; Global Health Innovation, Clinical, and Operational Research Division, Qure.ai Technologies Pvt Ltd, Bengaluru, Karnataka, 560042, India; Product Division, Qure.ai Technologies Inc., New York City, New York, 10281, United States; Global Health Innovation, Clinical, and Operational Research Division, Qure.ai Technologies Pvt Ltd, Bengaluru, Karnataka, 560042, India; Global Health Innovation, Clinical, and Operational Research Division, Qure.ai Technologies Pvt Ltd, Bengaluru, Karnataka, 560042, India

**Keywords:** low- and middle-income countries, artificial intelligence, chest X-ray, tuberculosis, medical imaging, implementation

## Abstract

Medical imaging is fundamental to healthcare systems, aiding in the detection of internal abnormalities related to medical conditions beyond their physical symptoms. In low- and middle-income countries (LMICs), limited access to advanced imaging and scarcity of radiologists for image interpretation are evident. Upgrading available resources with artificial intelligence can expand the diagnostic capacity of LMICs to manage the growing prevalence and incidence of infectious diseases such as tuberculosis (TB). Chest X-rays can act as an effective triage tool for TB screening, and multiple models have been reported to improve the number of cases detected in high-burden settings. The case-finding strategies reported in literature have also demonstrated improved diagnostic accuracy and turnaround time post adoption of artificial intelligence (AI) for chest X-ray interpretation. AI assistance can help in identifying radiological involvement of TB, irrespective of their clinical symptoms. Furthermore, cost-effective, integrated workflows can also efficiently support LMICs by facilitating parallel diagnosis and appropriate linkage to care for multiple chest disorders through a unified pathway, thereby broadening the capabilities of chest X-ray based TB screening. By optimizing and strengthening LMIC health systems with AI, further scale-up and implementation can foster a supportive ecosystem for early disease diagnosis and decentralized care delivery.

## Introduction

Medical imaging plays an indispensable role in healthcare systems around the world,^1^ transforming the way diseases are diagnosed and managed.^2^^,^^3^ From exclusive internal visualization of anatomies, prominent imaging technologies like X-ray radiography, computed tomography (CT), magnetic resonance imaging (MRI), ultrasonography, endoscopy, etc., have also expanded their capabilities toward early, accurate diagnosis, providing predictive insights on patient health for targeted interventions, therapeutic efficacy monitoring, etc., further impacting patient outcomes.^3^ Technological advancements in imaging have enabled the early detection of multiple diseases with minimal diagnostic errors and have provided deeper insights into internal and external abnormalities underlying a patient’s condition.^4^

### Overview of imaging challenges and gaps in LMICs

Globally, the radiological imaging ecosystem encompasses high-quality imaging modalities, trained resources, and digital information systems to accelerate radiology workflows. However, differences in resource allocation between high- and low-income nations overlook the distinct needs and impact on healthcare delivery.^5^ It is reported that two-thirds of the global population lack access to imaging services,^6^ leading to an estimated 2.46 million deaths.^5^ The disparity in access to advanced imaging technologies often results in challenges for accurate diagnosis and treatment planning in resource-constrained settings.^7^ An estimated 3.21 billion people in low- and middle-income countries (LMICs) lack even fundamental imaging services.^8^ The availability of cutting-edge imaging equipment and radiological expertise in LMICs (with 1 CT scanner, 1.12 MRI units, and 1.9 radiologists per million population) is significantly lower when compared to high-income countries (with 40 CT scanners, 26.53 MRI units, and 97.9 radiologists per million population).^9^^,^^10^ The inequities typically extend beyond availability of equipment to also restricting access due to longer travel distances and prolonged wait times for underserved populations.^11^ Furthermore, apart from widening disparities in access, poorly adapted technology transfer from high-income countries, inadequate device maintenance, and challenges in deploying advanced imaging technologies in LMICs raise significant concerns. In high-income settings, the evident uptake and successful implementation of imaging technologies have demonstrated improved health outcomes.^12^ These challenges impacting access to imaging technologies between countries are to be tackled to meet the universal health coverage and sustainable development goals by 2035.^9^

In LMICs, the increasing prevalence and incidence of communicable and non-communicable diseases (NCDs) have also emphasized the inevitable need for imaging in healthcare delivery to support diagnosis and treatment.^9^ The co-existence of infectious diseases with chronic non-communicable conditions like diabetes, hypertension, cardiovascular diseases, cancer, etc. has led to multi-morbidity and mortality, presenting a key public health challenge.^13^ Early diagnosis is pivotal to addressing this challenge by triaging the patients at risk requiring treatment, which has led to the frequent and routine use of point-of-care imaging modalities in clinical practice for diagnosis and disease management.^8^ Furthermore, to improve health outcomes in LMICs, increased innovations and investments in imaging technologies are required to specifically address local healthcare needs, geographical barriers, infrastructural limitations, resource adoption, alignment with end-user skills, etc. Low-cost, portable imaging technologies developed using local resources are essential for sustainable population-based healthcare in such environments.^12^

### Prevalent imaging modalities in LMICs for diagnosing diseases

Ultrasonography and X-ray radiography are the primary medical imaging modalities used in LMIC settings, and they help diagnose and address 70%-80% of illnesses.^8^^,^^14^ Low-cost, portable imaging technologies like digital X-ray systems and handheld ultrasound devices are highly recognized and known to be beneficial in such countries.^12^ However, technological upgradation is necessary to obtain higher diagnostic accuracy and sensitivity for diagnosis, and this continuous refinement has also made them more compact, lightweight, portable, inexpensive, and point-of-care. Factors like global internet access, up-to-date digital infrastructure, uninterrupted power supply, and breakthroughs in artificial intelligence (AI) have positively impacted imaging operations and facilitated automated image interpretation.^8^

As a widely adopted imaging technology in LMICs, non-invasive portable ultrasound is used to diagnose pneumonia and is known for its cost-effectiveness, real-time imaging, use of a minimally trained, non-specialized workforce for operation, and low maintenance.^9^^,^^15–17^ Point-of-care ultrasound has also been potentially used to identify sonographic signs of TB in children and to assist clinicians in overcoming the present diagnostic gaps in pediatric TB management. However, TB-specific lung cavities and consolidation are more definitively identified in chest X-rays (CXRs).^18^

Plain X-rays, or radiographs, are a conventional and pioneering diagnostic modality^19^ that is globally used to diagnose a wide range of pathological abnormalities like fractures, osteoporosis, cancer, dental infections, etc.^20^^,^^21^ Chest X-rays (CXRs) are the most performed radiological evaluation^22^ in primary healthcare settings of LMICs to typically diagnose respiratory infections like pneumonia and tuberculosis (TB).^19^ Due to its relatively low radiation exposure, CXRs are also used in early-stage lung cancer screening to improve survival^23^ and in identifying musculoskeletal trauma, bone fractures, dislocations, soft tissue injuries, etc.^24^

### Incidence of infectious diseases in LMICs and triage by CXR

TB is a treatable infectious disease affecting the world, with over 10.8 million active TB cases and 1.25 million deaths.^25^ Furthermore, the global interruptions in diagnosis, treatment, and care delivery of TB due to the COVID-19 pandemic are estimated to cause an additional 1.4 million deaths between 2020 and 2025, impacting the End TB efforts.^26^ It also reversed 12 years of the progress made by the global TB control and management initiatives.^27^ As of 2023, the number of undiagnosed or missed cases is reported to be 2.7 million, which is about 35% lower than the previous years. This gap between global TB incidence and the number of newly reported cases should be further reduced to meet the End TB goals (Table 1) worldwide.^30^ Thirty LMIC countries are responsible for 99% of newly diagnosed cases in 2023 and 98% of the global TB burden. India, Indonesia, China, the Philippines, and Pakistan contribute to a major 56% of the TB burden. TB affects the LMIC population disproportionately,^31^^,^^32^ raising challenges to their public health efforts.

**Table 1.** The global End TB indicators, targets, milestones, and corresponding strategy pillars of the End TB strategy^28^^,^^29^.

Global end TB impact indicators	Targets (from 2015 baseline)	Milestones (2015 to 2023)	Strategy pillars
TB deaths	95% reduction	33% reduction from 1.5 to 1.1 million	Integrated, patient-centred care and prevention include early diagnosis, systematic screening of high-risk groups and contacts, preventative treatment, etc.
TB incidence rate	90% reduction	9.88% reduction from 243 to 219 per 10 000 population	Intensified research and innovation enable development and rapid uptake of new innovative tools, impact interventions and implementation strategies
Catastrophic costs due to TB	0	Zero TB treatment incurred costs	Bold policies and supportive systems provide adequate resources for TB care and prevention
TB treatment coverage	≥90%	46.84% increase from 4.91 to 7.21 million people covered for treatment	Universal health coverage policy ensures treatment coverage for all notified cases via quality and rational use of medicines, and infection control

CXRs are notably used as an initial triage or screening tool for TB globally,^9^ and they identify at-risk individuals regardless of their clinical symptoms, help rule out normal cases and indicate who requires further confirmatory testing for diagnosis. The high sensitivity of CXR as an index test, both with and without concurrent symptom assessment, points to their potential for obtaining a high yield of TB cases.^33^ As per the technology landscape report 2021, CXRs identified 89% of microbiologically confirmed TB patients when CXR was used as a parallel case-finding tool along with conventional symptomatic screening. It then emphasized that 36%-80% of microbiologically positive TB cases did not manifest any clinical symptoms or were unaware of their symptoms.^34^ Finding missing TB cases with no reported symptoms is essential to control the spread of disease, and CXR can play a vital role in active case finding and community-based screening programs. Further, in the TB triage algorithm devised by the World Health Organization (WHO), CXRs also assist in the clinical diagnosis of bacteriologically negative TB cases. It can also optimize the use of expensive Xpert MTB/RIF assays by increasing the pre-test probability and positive predictive value of such test methods.^35^ LMICs contribute 95% of the reported TB deaths, and their co-existence with prevalent NCDs further impacts the population health outcomes, revealing the need for integrated health delivery models.^10^

### X-ray technology evolution and AI-enabled interpretation

In X-rays, technological innovations like digitization and the development of advanced detectors have contributed to the betterment of system processes such as image acquisition, interpretation, storage, and transmission. Modern, compact X-ray systems are also ultra-portable, providing equitable access to care by making screening of vulnerable populations in remote communities feasible, evading barriers.^36^ The costs involved in mass CXR screening are also plummeting because of digitization and integration of computer-aided detection in modern X-ray systems.^37^ Digitalization of X-ray detectors has also enabled cloud-based image storage, remote post-processing, and rapid image interpretation using artificial intelligence. Designed for resource-limited environments, these battery-operated, digital radiography systems can be transported in a backpack without the need for permanent infrastructure demands or staffing needs. Compatibility with Computer-Aided Detection (CAD) software further enables remote image transmission and interpretation to detect abnormalities, expanding the diagnostic capacities of CXRs in underserved areas and LMICs.^8^

Ultra-portable X-ray systems now make CXR screening accessible in hard-to-reach geographies, eliminating the need for any on-site or local health infrastructure. However, the scarcity of radiologists remains a critical challenge in LMICs, partly solved by integrating AI software with computer-aided detection that can interpret and report CXRs in a structured format. This further addresses the challenges of staff shortages, reporting delays, inter- or intra-reader variability, etc. CAD software has also proven to increase diagnostic performance among radiologists regardless of their expertise levels for identifying chest abnormalities like lung nodules, consolidation, pneumothorax, etc., and reduce CXR reading times.^22^ To summarize, decentralizing radiological interpretation and CXR screening using AI offers an opportunity to improve care delivery and outreach, potentially identifying missing TB cases.^38^

While CXR imaging presents challenges in interpretation, AI-assistance can reduce physician errors,^39^ improve the diagnostic accuracy of subtle findings, sort critical cases, and automate repetitive tasks like reporting. It can also assist emergency physicians and radiology trainees in the absence of expert senior radiologists by maintaining diagnostic efficiency and accuracy in decentralized settings. As reported in literature, AI assistance has increased sensitivity of thoracic abnormality detection by 26% and reduced reading times by 31%.^22^ In comparison to human reader-based traditional pathways, AI screening has exhibited a lower number needed to screen, dropout rate, and higher treatment coverage.^40^ The potential of AI to streamline real-world hospital workflows, reporting, and resource allocation to automate patient triaging was also reported in literature.^41^ Automated classification of normal CXRs can reduce imaging workload. With over 99% specificity in segregating abnormal CXRs, AI allows radiologists to focus more on critical cases.^42^ Along with high NPV (99%), by showcasing a satisfactory agreement with human readers, AI’s ability to classify CXRs in routine, high-volume settings were also noted.^43^

### AI in TB programs: global applications and evidence

Prevalence surveys in 33 African and Asian countries reported that over 70% of the TB-diagnosed individuals did not manifest any symptoms and were only triaged due to their radiological involvement. When more evidence demonstrated the significant impact of using AI software to accurately interpret CXRs and diagnose TB, WHO in 2016 revised TB screening guidelines, specifying the importance of using AI software as an alternative to human readers for digital CXR interpretation and triage of individuals above 15 years of age.^44^ Further, for AI-enhanced CXRs to be effective as a point-of-care triage tool for healthcare providers in ruling out and systematically screening for active TB, the WHO target product profile mandates a minimum of 90% sensitivity and 70% specificity.^45^ Globally, the Stop TB strategy by WHO emphasizes utilizing and empowering the existing infrastructure to control and eliminate TB. A holistic approach to TB control is pursued, and further endorsement and initiatives from the Stop TB Partnership to eliminate TB by 2050 and expect less than one case per million population also contribute to health system strengthening and addressing barriers of disease management.^46^^,^^47^

### Implementation experiences with CXR AI in TB case finding

#### Active case finding

Active case finding (ACF) for TB targeting high-risk populations includes strategies like mass CXR-based screening, home-based surveys, and facility- or community-based symptom screening.^48^ In high-risk settings with increased TB incidence, like prisons, mass screening using AI-based CXR is performed as a primary ACF strategy to tackle the disease burden and spread. Irrespective of their symptoms, the incarcerated population are systematically screened and tested for TB periodically.^49^ It is reported that such active case interventions using AI-based CXR minimize detection gaps impeding the spread of the disease among vulnerable populations.^50^ A meta-analysis of 7 CAD evaluations has reported a pooled sensitivity of 0.87 (0.78-0.96) and a pooled sensitivity of 0.74 (0.55-0.93) CAD for pulmonary TB detection, highlighting the capability of ACF implementation to reduce disease transmission among target population and changing TB epidemiology.^51^

#### Community outreach

**Figure 1.**
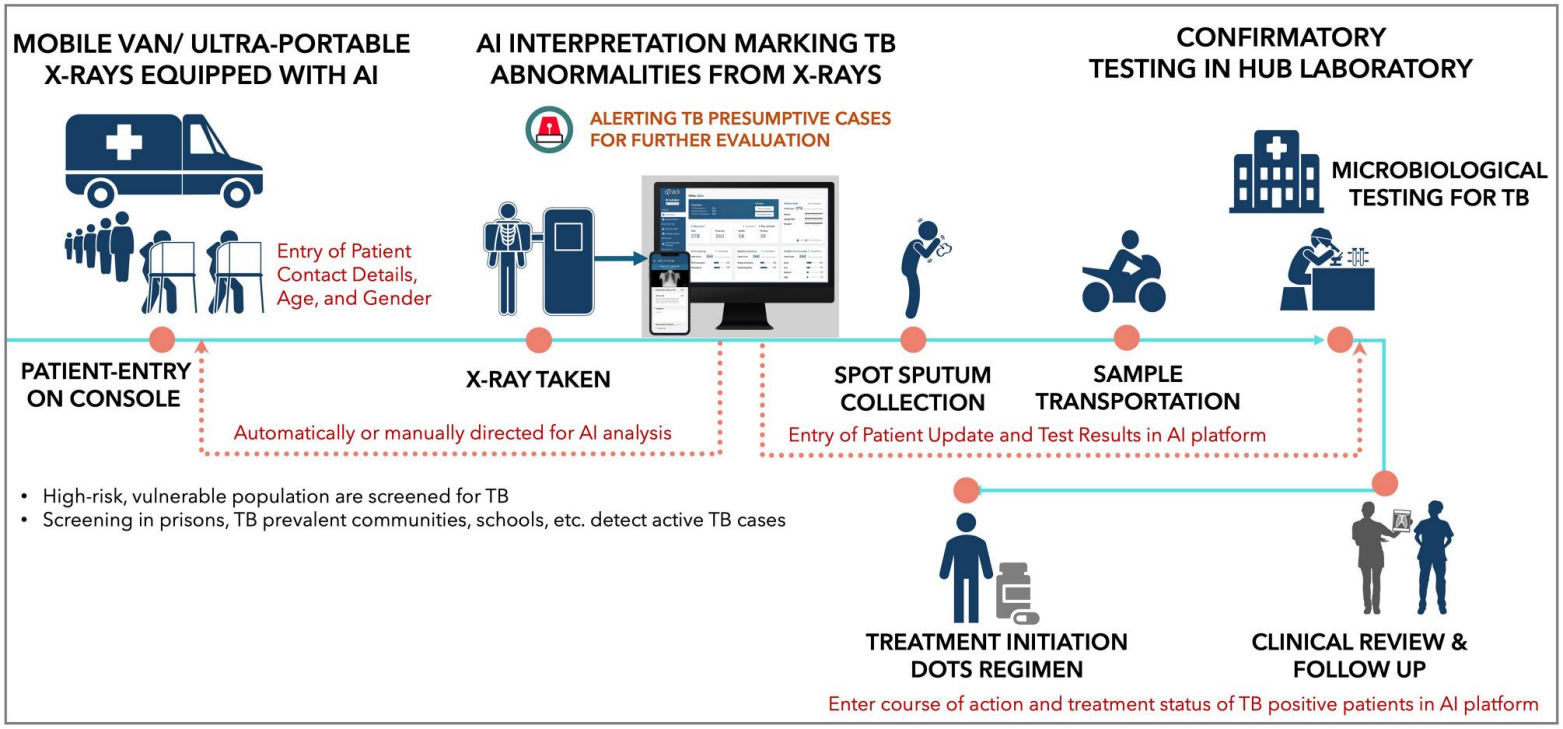
Active case finding in community settings using mobile van and AI based CXR interpretation.

Although modern X-ray machines mounted on mobile units are used for community outreach targeting TB, certain remote communities remain hard to access. To reach remote, rural populations in northeast Nigeria, ultraportable X-ray devices equipped with AI-based CXR interpretation were used in a reported study (workflow depicted in Figure 1). These devices can be easily transported to remote areas in small vehicles, and they can function without electricity. As the devices are integrated with CAD, it enables detection of TB cases irrespective of their symptoms and assist trained health workers to triage people for confirmatory testing.^52^ In African communities affected by HIV and prior TB, populations have atypical radiological presentation, an asymptomatic nature, and sputum scarcity. While intervening with ACF activities of TB, this impacts the accuracy of CAD, and stratification by subgroups to ensure optimal performance.^51^

#### Intensified case finding or TB surveillance model

**Figure 2.**
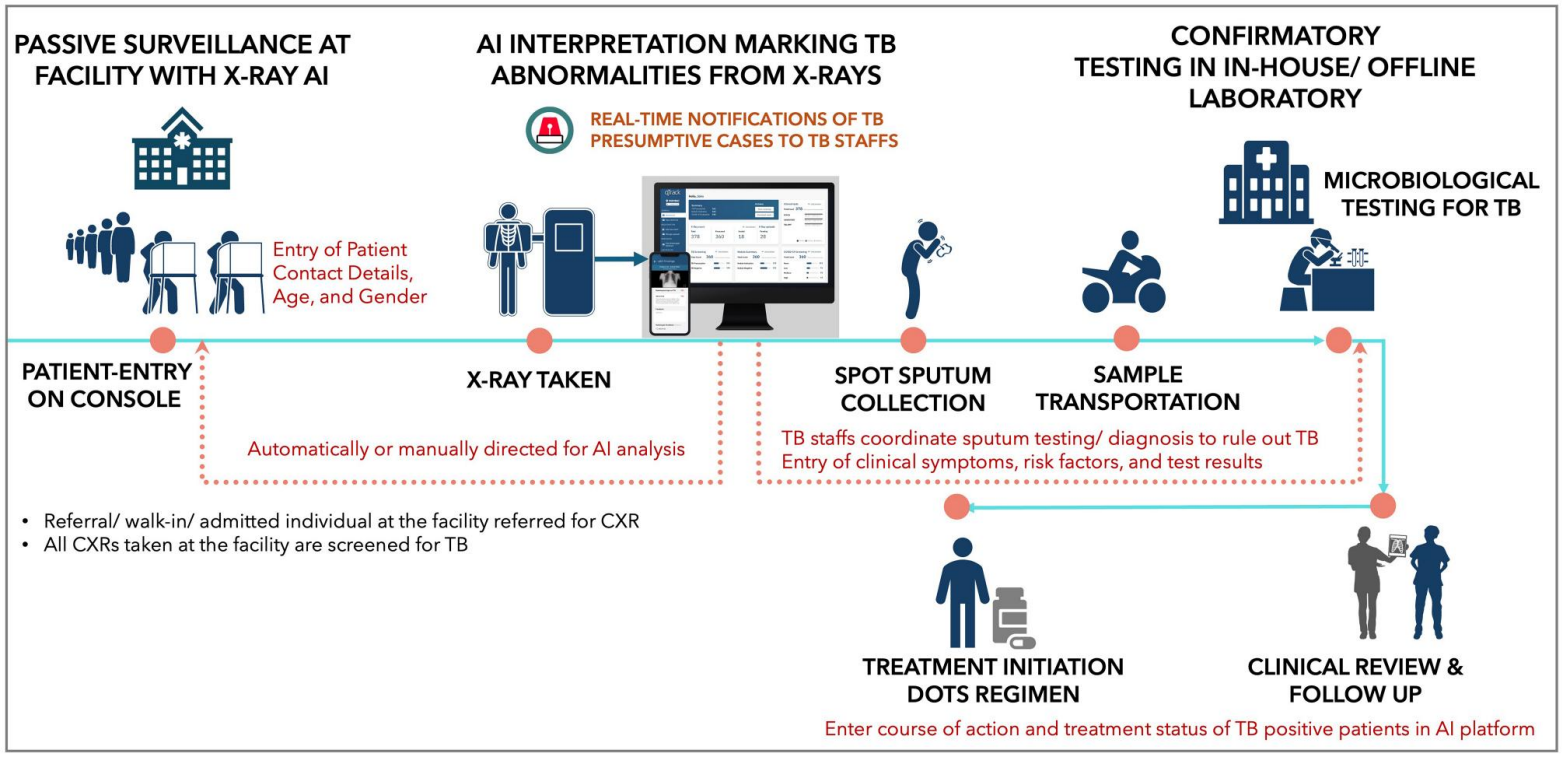
AI-augmented TB surveillance model in facilities for TB detection and management.

Passive case finding can detect active TB disease among symptomatic cases seeking care at facilities.^49^ However, detecting cases only after symptoms appear often results in greater disease severity. Hence, facility-based intensified case finding complements ACF and potentially captures more cases that are missed (workflow depicted in Figure 2). Relatively, this strategy requires fewer resources and less effort as it leverages the existing hospital infrastructure.^53^ Implementing parallel screening of all facility-acquired CXRs can function as an effective TB surveillance system, facilitating the early detection of individuals with TB signs or risk factors, irrespective of their presenting symptoms. Surveillance models incidentally detect presumptive cases of TB, who are further evaluated and managed through the TB workflow in the facility. Incidental detection includes identifying asymptomatic or subclinical TB cases, uncovering the otherwise missed cases.

### Challenges and learnings from LMIC deployments

Deploying AI in a resource-limited setting can cause real-life challenges related to infrastructural readiness, operational feasibility, acceptability of technology adoption, etc., that are to be addressed for a successful implementation (Table 2). Qualitative findings have led to devising strategies that ensured seamless installation, internet connectivity, communication with multiple stakeholders, etc. to address barriers in an ACF implementation in India. Parallelly alongside diagnostic accuracy and cost, AI implementation should also consider relevant features like operational requirements, deployment mechanisms, machine compatibility, regulatory clearances specific to geographies, data privacy, possibility of integration into existing health systems, etc..^45^^,^^60^ Continuous efforts to train and engage healthcare workers and communities, emphasizing the need for technology familiarity, adoption, and incorporation into healthcare systems, are also critical for scalable and sustainable implementations. Training initiatives hold the potential to enhance healthcare provider performance in LMICs, strengthening care delivery and positively impacting billions.^61^

**Table 2.** Summarization of the challenges and specific solutions adopted related to real-world LMIC deployments.

Factor	Challenges	Specific solutions and learnings	Reference
Threshold Modification	Pre-configured (one-size-fits-all) threshold may not be generally applicable as an optimal threshold can significantly vary across population and geographies.	Customizing threshold effectively dichotomize the binary system output and prompt confirmatory testing Local calibration enables the algorithm to achieve the WHO TPP Evaluation framework can help implementers with optimal threshold selection	^54^ ^,^ ^55^
Finding Missed Cases	Screening using symptoms may miss people with TB (50%), who can be bacteriologically confirmed.	Missed cases from symptom screening can be identified by AI-CXR screening with higher specificity. Screening combination of CXR and common TB symptoms demonstrate to improve the sensitivity of detecting active PTB, especially in people living with HIV.	^56^ ^,^ ^57^
Optimized use of Ultraportable systems	Ultraportable CXR systems with AI can screen remote and hard-to-reach population	Lower battery life by high-capacity power bank to aid screening throughout the day Operational delay with battery-operated system by externally powered portable reference system Slow wireless transfer of images via bluetooth connectivity by wired alternatives in workflow Lower image quality by AI-based evaluation and ALARA (As Low As Reasonably Achievable) dose efficiency.	^36^
Universal GeneXpert Testing Costs	AI-CXR triage to identify those who require microbiological testing	AI-based triage lowered projected costs, averted additional DALYs, and improved TB detection in low-income high-burden settings	^58^
Technical Compatibility	Challenges due to varying file format, internet connectivity, integration, deployment, etc.	Updating solution to support multiple digital file formats apart from DICOM Flexible models: One-System or Two-System setup for software installation and processing in workflows	^59^
Gap in Awareness	Reluctance toward technology adoption by the local radiology association and private facilities.	More awareness of AI and its regulatory clearance in the Indian context were disseminated over monthly meetings	^59^

With WHO’s advocacy of using AI for TB screening,^44^ an operational research toolkit was also designed to guide local calibration of CAD thresholds, along with estimating accuracy metrics, diagnostic costs, and prevalent cases detected, further enabling the effective use of CAD algorithms in TB detection.^62^ As the WHO guidance formally advised CAD as an alternative to human readers for TB screening since 2021, their evaluations assess whether AI could meet the WHO TPP. Despite global validation and recommendations, additional regulatory compliance with any national policy governing the use of CAD software for TB screening and triage is necessary for its adoption. Authorized approval for importation, inspection, and radiation safety from the Radiation or Nuclear Authority or the Atomic Energy Regulatory Authority are required for use. In-country local demonstration of CAD software reading can help to select appropriate operating points. Further, data privacy risks and concerns also demand data storage and transfers in the cloud or servers locally within the country of implementation.^63^

It is to be noted that AI offers a cost-effective alternative to radiologists for large-scale TB screening programs in high-burden countries. For cost considerations, target screening numbers, AI software licenses, performance, and operational features are assessed. Integrating CAD software into case-finding can be economical,^64^ and multiple retrospective studies have reported its effectiveness in reducing the use of Xpert MTB/RIF tests by over 60% without compromising the sensitivity (>90%).^54^^,^^55^ A cost-effectiveness analysis funded by the United States Agency for International Development (USAID) has revealed a lesser incremental cost-effectiveness ratio of Philippine Peso (PHP) 43 376 per disability-adjusted life-years (DALY) for AI-powered CXR screening compared to human readers (PHP 47 667).^65^ By identifying an additional 1197 TB cases and preventing 55 deaths over 10 years, the incremental costs per facility by AI have lowered to PHP 76.8 million, a 9% decrease from that of human readers (PHP 84.4 million). Further, a health technology assessment in India also identified AI as a dominant cost-saving strategy, with an ICER of INR −9864.77 per case detected, making it more effective and cheaper than routine care.^66^ In comparison to other diagnostic tests like upfront smear microscopy and GeneXpert, AI-based triage was projected to lower costs by 19% and 37% while averting 3%-4% and 4% more DALYs, respectively.^58^ The impact of AI integration with respect to public health begins with addressing access barriers that further lead to improving diagnostic yield and cost savings and finally streamlining workflow and time to diagnosis.^44^

### Expanding the scope of imaging: beyond TB

Beyond TB, AI systems can identify various chest abnormalities related to the heart and lungs. These clinically relevant findings can aid in diagnosing conditions like cardiomegaly and pulmonary diseases, facilitating appropriate linkage to care.^67^ Population-based mass CXR screening often identifies potential diseases and abnormalities beyond TB, and it presents an opportunity for multi-disease integrated health screening and management. In LMICs, the growing prevalence of NCDs like cardiac disorders, chronic respiratory conditions, and cancer necessitates the integration of clear referral pathways, diagnostics, and linkage to care for non-TB pathologies within ACF and TB screening programs.^68^ A community-level implementation study has reported greater prevalence of cardiomegaly among people with co-existing NCDs and the need for interventions to manage cardiovascular NCDs at primary care levels.^69^ Similarly, lung cancer is common in TB-prevalent settings, mimicking clinical manifestations that can result in potential misdiagnosis. Hence, with AI assistance, additional detection of lung cancer cases within existing TB workflows is feasible and presents the potential to scale up screening for other NCDs, improving patient outcomes in LMICs with lesser incremental cost. Feasibility studies incorporating AI-based lung nodule detection in TB-endemic settings have also resulted in diagnosis (47.1%) and treatment (64.8%) of lung cancer patients, post-CT scan referral, and follow-up.^70^

### Call to action: innovations and sustainable uptake

**Figure 3.**
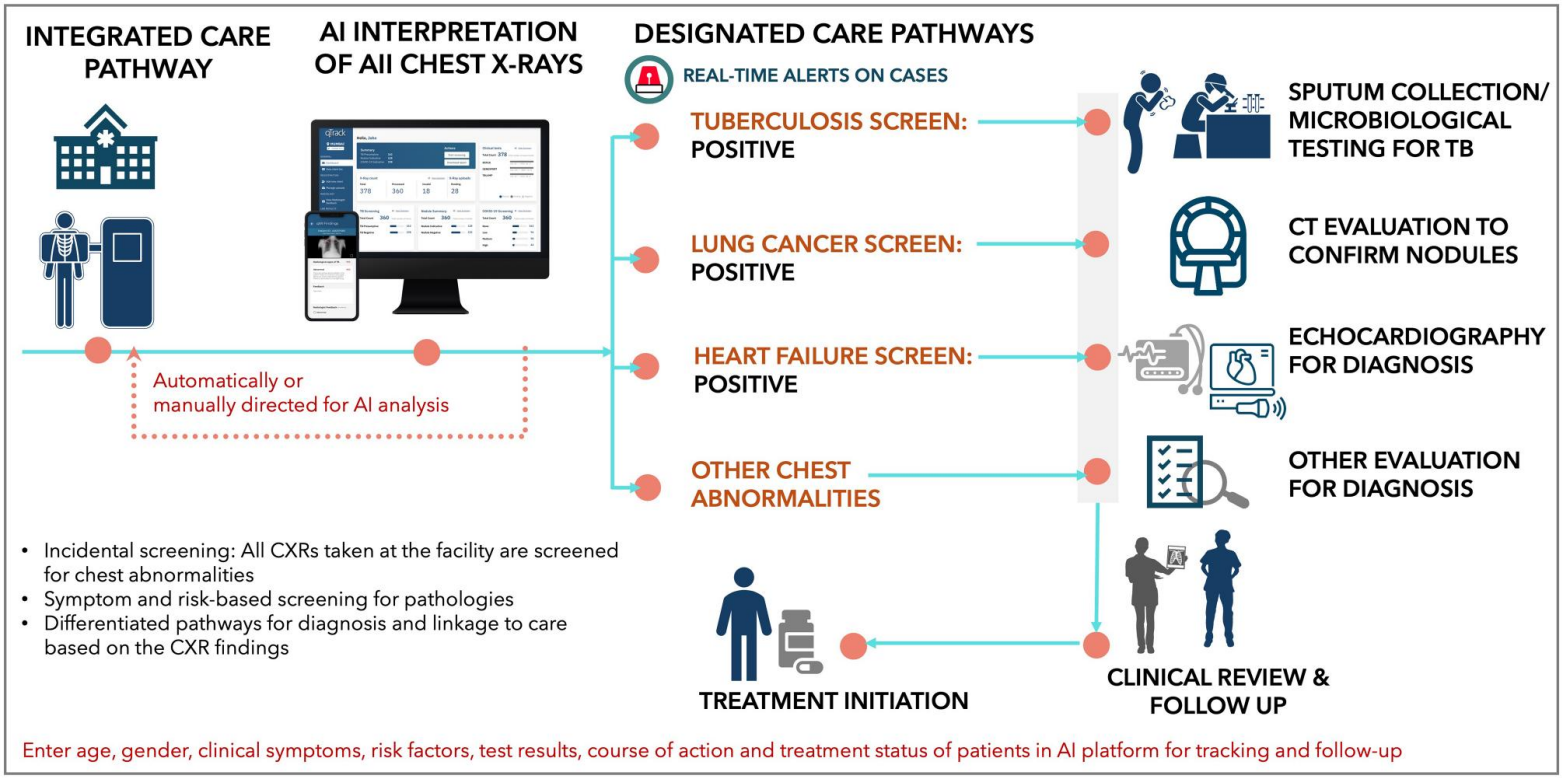
AI enabled integrated health screening and management pathway for disease diagnosis.

The WHO’s Practical Approach to Lung Health initiative aims to streamline and integrate lung care delivery—including screening, classification, and frontline worker coordination—using digital technologies. This integration can mitigate misdiagnosis in case of overlapped manifestation of diseases and define clear guidelines for case management, including minimum diagnostic criteria and severity assessment. Integrating TB and NCD services can also effectively address their combined burden in LMICs.^71^ AI-powered technologies have the potential to revolutionize integrated lung screening pathways by addressing critical clinical and operational challenges like diagnostic errors, reporting delays, and lack of multi-stakeholder coordination in resource-constrained settings. Detecting multiple chest abnormalities alongside TB in facilities (as shown in Figure 3) and decentralizing care delivery and expanding access using mobile devices can allow sustainable integration and strengthening of healthcare systems.

## Conclusion

The evidence reported so far unequivocally supports the integration of AI with CXR analysis in critical high-burden applications like TB and multi-disease diagnosis. However, the need for scale-up in successful implementation is real, and it can be streamlined by aligning with the digital health roadmaps and strategic goals of LMICs in disease management. As per Universal Health Coverage 2030, AI use promises significant cost efficiencies and accelerated expansion of healthcare access. Looking ahead, the regulatory ecosystem should also keep pace with the evolution of technological innovations. With the advent of generative AI and large language models, AI-derived insights and reports can now support frontline clinical decision-making and decentralize care delivery, ultimately offering a powerful pathway to optimize limited health system resources.
